# Safety considerations in the evolving legal landscape of psychedelic-assisted psychotherapy

**DOI:** 10.1186/s13011-022-00468-0

**Published:** 2022-05-14

**Authors:** Victor Mocanu, Lindsay Mackay, Devon Christie, Elena Argento

**Affiliations:** 1 Department of Medicine, University of British Columbia, British Columbia Centre on Substance Use, 400-1045 Howe St, Vancouver, BC V6Z 2A9 Canada; 2 Department of Medicine, University of British Columbia, 2255 Wesbrook Mall, Vancouver, BC V6T 2A1 Canada

**Keywords:** Health policy, Compassionate use, Exemption, Psychedelics, Psilocybin, MDMA, Canada

## Abstract

International drug policy is rapidly evolving in tandem with promising evidence for psychedelic-assisted psychotherapy (PAP) in treating a range of mental health conditions. Canada is among the countries increasingly expanding access to psychedelic substances for therapeutic purposes. The 8-year ban on medical exemptions through the Canadian Special Access Programme was recently reversed in January 2022 and the first exemptions for legal possession and personal use of psilocybin mushrooms were granted in 2020, nearly 50 years since their criminalization. In view of the evolving evidence base and regulatory landscape for PAP illustrated by recent shifts in Canadian and international drug policy, this piece seeks to clarify the special range of factors which ought to be considered to safely expand access to psychedelics. Streamlining access to safe and evidence-based compassionate use of PAP will provide a timely treatment option to those currently in need while encouraging further research and outcome surveillance to refine best practices.

## Background

Psychedelics are receiving increasing attention from the public, clinicians, and policymakers as evidence mounts to support psychedelic-assisted psychotherapy (PAP) in treating a range of mental health conditions and addressing a large unmet need. From a historical perspective, Indigenous peoples have used naturally occurring psychedelics such as *Psilocybe mexicana* mushrooms in ceremonial, ritual, and healing practices for centuries [1, 2], whereas PAP is a relatively recent development in Western biomedical knowledge. Lysergic acid diethylamide (LSD) and 3,4-methylenedioxymethamphetamine (MDMA) were first synthesized in the early twentieth century while psilocybin was isolated from *P. mexicana* and entirely synthetically produced by the early 1960s [2]. Psychedelic research in the 1950–60s began to demonstrate promising results with these serotoninergic agents before coming to a halt in the 1970s due to criminalization, falling short of translation into more widespread therapeutic use [1–3]. A combination of factors motivated the policy shift including notorious examples of unethical experimentation (e.g. LSD in MK-ULTRA), concern for overeager public use outstripping the available evidence on benefits and harms, particularly by the counterculture of the time, and contemporary anti-drug politics with the launch of the “war on drugs” [1, 3, 4].

The recent revival of international psychedelic research has re-emphasized the therapeutic potential of PAP as a novel treatment approach in the context of an increasing burden of refractory mental health conditions and limitations to currently available treatment options. For example, randomized control trials (RCTs) have found clinically relevant reductions in depression and anxiety symptoms for the majority of PAP participants with cancer-related distress [2], effects which can endure for years [5]. A recent multinational phase 3 placebo-controlled RCT provides encouraging results for MDMA-assisted psychotherapy for post-traumatic stress disorder (PTSD) [6], and psilocybin achieved comparable outcomes to escitalopram as a psychotherapy adjunct for major depressive disorder in a phase 2 RCT [7]. Preliminary findings also signal benefit for individuals with substance use disorders [8, 9], for which several RCTs are currently underway (ClinicalTrials.gov). Qualitative studies suggest that participants derive these health benefits through rich, highly personal, and often spiritual psychedelic experiences which afford new insight into their disease and illness experience [10].

Current evidence further indicates an impressive safety profile of PAP when delivered in carefully constructed and monitored settings. Clinical trials have demonstrated efficacy and found no serious or persistent adverse effects when selected participants receive psychedelics to augment longitudinal psychotherapy with trained practitioners in a supportive environment [1–3]. The specialized design and delivery of PAP in landmark studies should underscore the modern understanding that potential health benefits of psychedelics are not merely derived from their pharmacology. Positive treatment outcomes are influenced in large part by the broader context of psychedelic substance use and subjective experience in a supportive psychotherapeutic setting [11].

Considering the history and growing evidence base of PAP, this piece will examine recent shifts in psychedelic drug policy both in Canada and internationally. Evidence-based practice and participant safety are paramount for the cautious reintroduction of psychedelics into clinical research and practice. Informed of these standards and safeguards, several practical recommendations are provided which can be integrated into access mechanisms to facilitate safe, timely access to evidence-based PAP.

## Main text

### Pre-approval access pathways for PAP

Prior to formal regulatory approval for medical uses or decriminalization, certain pathways exist to legally access psychedelics via legal exemptions for research, personal use, or medical purposes. These exemptions can allow individuals with serious or life-threatening conditions the “right to try” a particular treatment which is restricted in their respective country or jurisdiction. Research exemptions are designed to facilitate rigorous investigation of drug efficacy and safety which incurs financial and labour costs, limiting the number of people obtaining access through this route. Exemptions for personal use simply allow for the individual to legally possess and use a controlled substance without strict requirements for setting, administration, or professional guidance, although therapeutic intent may often be claimed to support the legitimacy of the application. In this view, exemptions for personal use might be the less cumbersome relative to research exemptions but more liable to non-evidence-based use with more lenient parameters, particularly where psychedelics may be used outside of psychotherapeutic settings in which efficacy is proven, and furthermore may miss the opportunity to report valuable data to inform further research, clinical, or public use. Lastly, medical exemptions may be granted when practitioners submit an application to supervise patients receiving the restricted treatment, provided evidence that other treatment options are exhausted or illness is particularly severe, and subsequently report health outcomes. As such, medical exemptions offer a compromise between exemptions for research and personal use whereby access is relatively more streamlined than research applications but mandate more monitoring of patient safety and outcomes as compared to exemptions for personal use.

As psychedelics remain scheduled under the Controlled Drugs and Substances Act (CDSA) in Canada, recent efforts to expand legal access have focused on the avenues of exemptions for research, medical, and personal use purposes. While a small number of Canadians obtained research exemptions to use MDMA for PAP in the recent positive PTSD trial [6], and more research exemptions can be expected as the field of psychedelic research grows, an unmet need will remain outside of clinical trials. Coming at an opportune time, the 8-year ban on medical exemptions for psychedelic substances through the Canadian Special Access Programme (SAP) was recently reversed in January 2022, restoring potential access through this route [12]. Historically, psychedelics and other controlled substances were collaterally banned in politicized 2013 amendments primarily targeting diacetylmorphine (prescribed heroin) [13, 14]. The measure was meant to prevent clinical trial participants who found benefit in expiring research exemptions for diacetylmorphine from continuing to access treatment via the SAP [14].

Although the SAP was shortly revised to re-enable requests for diacetylmorphine, the ban on applications for psychedelics remained until January 2022 when Health Canada reversed the 2013 amendments, citing overwhelmingly positive feedback received in a 60-day consultation period [13]. Notably, Health Canada clarifies that the SAP continues assessing requests for emergency use on a case-by-case basis without widespread or guaranteed access, although particular mental health professional qualifications or practices themselves are not strictly defined, and stipulates that manufacturers will need a valid dealer license under the CDSA [13]. Timing-wise, Health Canada aims to process SAP applications within two business days and expects practitioners will need 2 h or less to complete an application with another hour or less to report health outcomes following use [13].

In lieu of research exemptions and prior to amendments restoring access via the SAP, Canadians instead sought access through personal use exemptions from the CDSA, termed subsection 56 (1) exemptions. The first such exemptions to legally possess and use psilocybin without particular requirements for therapeutic process or practitioner guidance were granted in August 2020 to four individuals with end-of-life distress [15]. Since that time, subsection 56 (1) exemptions were granted to at least 60 Canadians with end-of-life distress, mood, anxiety, and substance use disorders as well as healthcare professionals training to provide PAP [16].

### Limitations of current access to PAP in Canada

Despite the growing body of evidence and favourable safety profile of PAP, safe legal access remains greatly limited in Canada. Subsection 56 (1) exemptions, although progressive in the context of modern drug policy, unfortunately proved difficult to obtain in a timely manner for urgent indications [16]. Wide unchecked provision of subsection 56 (1) exemptions with more leniency in terms of setting, administration, and professional guidance compared to medical exemptions could alternatively jeopardize best practices, participant safety, and hopes of further expanding PAP access in Canada.

As access pathways continue to shift, the current literature surrounding contextual and patient safety factors of PAP are essential considerations for policymakers and practitioners. In the Canadian experience, subsection 56 (1) exemptions highlight potential gaps in the safe and timely access to PAP which can likewise affect SAP applications and similar pre-approval mechanisms abroad. Aside the uncertainties of turnaround time and approval for both access pathways in Canada, there remains a paucity of training opportunities and qualified professionals to safely conduct research and potential future treatment with PAP, leaving healthcare providers and advocates to take responsibility for safely expanding access themselves [16].

An ongoing lack of necessary training and monitoring for PAP practitioners may pose serious safety risks for the public as individuals may seek treatment in settings or from therapists without the skills required to manage individuals while under the effects of psychedelics. Moreover, participants under the influence of psychedelic substance maybe be more vulnerable to predatory behaviour, misconduct, or other boundary issues among therapists which can seriously jeopardize not only patient health and safety, but public trust in the research and practice of the field at large [17]. Expert groups have begun addressing this issue by developing approaches to screen and train therapists with emphasis on codified ethics, practice standards and professional guidelines as similarly endorsed and enforced in other health professions [18]. Similar sustained efforts are urgently required in Canada not only for the training of providers but their monitoring in practice to support participant safety and trust.

### International examples of expanding access for PAP

In application, approvals for both research and medical use can complement each other, as in the case of MDMA-assisted psychotherapy for PTSD. Shortly after interim phase 2 data demonstrated large effect sizes for MDMA-assisted psychotherapy with 67% of participants no longer qualifying for the diagnosis of treatment-resistant PTSD at 1 year of follow-up, the United States (US) Food and Drug Administration (FDA) granted a “Breakthrough Therapy Designation” to expedite further development and regulatory review [19]. The FDA partnered with researchers to provide guidance on the ground-breaking positive phase 3 RCT of MDMA-assisted psychotherapy conducted in the US, Israel, and Canada [6]. The clinical experience at these research sites went on to attract medical exemptions from local policymakers in Israel and the US based on existing research protocols [19]. The Israeli Ministry of Health provided compassionate use approvals to 50 individuals with PTSD for MDMA-assisted psychotherapy in 2019 and shortly afterwards the FDA approved an Expanded Access program for another 50 individuals in the United States [19]. As the recent history of MDMA-assisted psychotherapy indicates, there is potential synergy between research and medical exemptions, when permitted by drug policy, to advance both clinical research and compassionate use.

International examples also raise the possibility of expanding access to PAP through decriminalization and legalization of psychedelic drugs. In addition to benefits rendered for the physical, psychological, and social safety of individuals using illicit psychedelics at risk of punishment by law, decriminalizing or legalizing psychedelics can also facilitate discourse and legislation needed to implement PAP itself. For example, the 2020 Oregon Measure 109 (Psilocybin Services Act) made impressive legislative reform for the state health agency to begin legal manufacturing, delivery, and supervised administration of psilocybin products at state-licensed programs by 2023 [20]. Notably, the legislation does not require specific diagnosis to use psilocybin nor does it strictly require psychotherapy to accompany psilocybin use [20]. Measure 109 does however signal other regulatory measures resembling PAP such as establishing training and practice parameters for licensed providers, developing guidelines for preparatory and administration sessions, and committing to ongoing regulation of psilocybin services [20].

As Measure 109 illustrates, a coordinated systems-level effort to expand and regulate legal access to psilocybin can transform access to PAP. Alongside dozens of legislative reforms to decriminalize or legalize psychedelics across the US, including in Oregon where all drugs have been decriminalized, international examples have generated interest to decriminalize psychedelics in Canada with a framework based on existing cannabis regulations [21]. The decriminalization strategy alone does however present drawbacks including the risk of overenthusiastic or politicized use of psychedelics from informal or formal distribution channels which may not be informed by best available evidence and arguably contributed to initial backlash and criminalization of psychedelics decades ago [1, 3, 4].

### Future directions for reintroducing and scaling PAP

As evidence accrues and access to PAP improves, Canadians welcome the recent SAP amendments to reinstate emergency medical exemptions for psychedelics [13]. Nevertheless, a calculated approach is essential to translate rapidly evolving evidence into best practices and keep the “psychedelic renaissance on track.” [4] On a systems level, similar to the mandate of the Oregon Psilocybin Services Act, coordinated efforts are needed to establish regulating authority to define and evaluate PAP including patient safety and practice standards [22]. In this pursuit, capacity building in training and licensing is urgently needed [23], especially as demands increase with broader implementation of PAP.

Regarding the recently amended SAP, a similar awareness of current evidence, patient safety factors, and practice standards is required in reviewing applications for psychedelics. Among these practice standards should be the manualized training and credentialization of Canadian mental health professionals requesting psychedelics through the SAP. Given the specialized knowledge and skills needed to deliver PAP, applications should be assessed accordingly for provider credentials such as the completion of a recognized training curriculum as is currently offered in several Canadian settings [24, 25], which potentially involves firsthand PAP experience via subsection 56 (1) exemptions for therapists to better empathize with psychedelic experiences of their patients [26]. Training programs for PAP providers, like the field of PAP itself, are novel and evolving rapidly to establish accredited curricula and practice standards for adequately training qualified providers, which remains a bottleneck for the safe and effective delivery of PAP. Recent amendments to the SAP and other such exemption mechanisms internationally provide further opportunities to develop training requirements set by established training programs [18]. Furthermore, SAP applications should indicate participant selection according to clear evidence-based indications (e.g. PTSD, end-of-life anxiety or mood disorder) and contraindications (e.g. no personal of family history of psychosis) as supported by modern primary literature [2]. The evidence base can further serve to inform drafting and review of SAP applications with a clear, thoughtful process proposed for administering PAP involving preparatory and integration sessions of conventional psychotherapy delivered in a controlled, safe, and supportive environment [2, 6, 7]. Given the same foundational evidence base, similar criteria likely translate for the adjudication of special access requests for evidence-based PAP in other international contexts. Finally, the SAP obligation to report clinical status, outcomes, and adverse reactions in follow-up of PAP will provide a valuable measure for monitoring patient safety in these circumstances.

Illustrated by the increasing range of indications and psychedelic substances used in different positive PAP trials, a certain degree of flexibility exists in terms of what exactly constitutes a supportive setting and process of conventional psychotherapy. Some variability between different practitioners and programs within Canada and internationally will be inevitable and well-tolerated so long as participants can engage safely and find benefit. This adaptability will likely present a strength in the implementation of PAP for diverse settings and populations provided that similar standards and safeguards are maintained such as provider credentialization, evidence-based practice of PAP, and outcomes monitoring. Moving forwards, pragmatic clinical trials, complementary observational and qualitative studies, and clinical outcomes surveillance will be invaluable to ensure PAP in real-world application is indeed benefitting participants and reassure initiatives in quality improvement and access expansion [27].

## Conclusions

Despite the current challenges faced in accessing PAP, the growing body of evidence and evolving legal landscape both in Canada and internationally are causes for cautious optimism. Several actionable targets exist for health advocates and policymakers to expand safe and timely legal access to PAP. One aim should be the evidence-based design and appraisal of applications for pre-approval use such as compassionate use medical exemptions via the recently amended Canadian SAP. As well, coordinated efforts in capacity building and regulation are needed to expand formal training, establish practice standards, and begin monitoring clinical outcomes as PAP is increasingly implemented. In the timely reintroduction of psychedelics into clinical research and practice, the standards and safeguards of PAP cannot afford to be overlooked.

## Data Availability

Not applicable.

## Abbreviations

PAP: Psychedelic-assisted psychotherapy
SAP: Special Access Program
LSD: Lysergic acid diethylamide
MDMA: 3,4-methylenedioxymethamphetamine
RCT: Randomized control trial
PTSD: Post-traumatic stress disorder
CDSA: Controlled Drugs and Substances Act
US: United States
FDA: Food and Drug Administration
